# Exploring Bacterial Communities and Functions in Phytophagous *Halyomorpha halys* and Predatory *Arma chinensis*

**DOI:** 10.3390/insects16020146

**Published:** 2025-02-01

**Authors:** Hongmei Cheng, Xiaoyu Yan, Changjin Lin, Yu Chen, Le Ma, Luyao Fu, Xiaolin Dong, Chenxi Liu

**Affiliations:** 1 Sino-American Biological Control Laboratory, Institute of Plant Protection, Chinese Academy of Agricultural Sciences, Beijing 100193, China; chenghongmei1010@163.com (H.C.); linchangjin163@163.com (C.L.); fuluyao5989@163.com (L.F.); 2 Department of Entomology, Yangtze University, Jingzhou 434023, China; yanxiaoyu0316@163.com (X.Y.); chenyu2632@163.com (Y.C.); male0402@163.com (L.M.); dongxl@yangtzeu.edu.cn (X.D.)

**Keywords:** microbiota composition, feeding, digestion, reproduction, biological control

## Abstract

The phytophagous *Halyomorpha halys* (Hemiptera: Pentatomidae) is a global agricultural pest that damages many crops. Conversely, the predatory *Arma chinensis* (Hemiptera: Pentatomidae) shows promise as a biological control agent against lepidopteran and coleopteran pests. *Halyomorpha halys* and *A. chinensis* are closely related species with different feeding habits, as confirmed via genomic and morphological analyses. However, no study investigating the implications of these differences has been reported. Herein, 16S rRNA sequencing technology was employed to analyze the microbiota diversity and function in different tissues (salivary glands, gut, sperm, and ovaries) of *H. halys* and *A. chinensis* to elucidate these differences from a microbial perspective. Additionally, the adult male-to-female ratio in *A. chinensis* organs was statistically similar, while that in *H. halys* was not. Based on the dominance of the symbionts in the two bug species, we inferred that *Sodalis* is involved in reproduction and digestion in *A. chinensis*, while *Spiroplasma* and *Pantoea* play essential roles in *H. halys* reproduction and digestion. We analyzed the data on the microbial diversity of two bug species, laying a foundation for further understanding microbial symbiosis in *A. chinensis* and *H. halys*, which may inform the development of biological control strategies.

## Simple Summary

Symbiotic bacteria play a crucial role in insects, exerting a profound impact on the phenotype and behavior of the host. *Halyomorpha halys* and *Arma chinensis* are closely related species of Pentatomidae with different feeding habits. Bacteria play a pivotal role in their behavior. Our investigation into microbial diversity within the feeding, digestive, and reproductive organs of the two species revealed significant differences across various organs. Notably, the gut, a vital digestive organ, harbors a more diverse array of microorganisms. Furthermore, bacteria present in the testes may have served as a critical driver in the genetic evolution of both species. Additionally, predatory *A. chinensis* exhibits an even more intricate bacterial diversity.

## 1. Introduction

Stink bugs (Pentatomidae) are a family within Heteroptera comprising plant feeders and predators in the suborder Hemiptera [1], including agricultural pests and pest natural enemies of great economic benefit to the agricultural industry [2,3]. The phytophagous *Halyomorpha halys* is a Pentatomidae pest of several globally important crops [4,5] that feed on the sap of vegetables, fruit, beans, and grains. The damage caused by *H. halys* is typical of pentatomids and is aggravated by the pest’s polyphagy and by the behavior of adults, which move continuously from plant to plant, from hedges or herbaceous crops to fruit orchards. The predatory *Arma chinensis* (Pentatomidae) is a promising biological control agent that preys on agricultural and forestry lepidopteran and coleopteran pests [6,7]. It can be easily mass-reared using artificial diets or its preys and exhibits strong adaptability to diverse ecological niches, enabling its successful and widespread use. *Halyomorpha halys* and *A. chinensis* are closely related species based on genome sequencing results [8,9] and morphological classification [10,11], but have opposite feeding characteristics.

Through long-term evolution, insects have formed interdependent symbiotic relationships with various microorganisms [12,13,14]. Endosymbionts provide nutrients to the insect host [15,16,17], expand the food range of the host [18,19], regulate host behavior and reproduction [20], participate in host metabolism [21], provide protective defense [22,23], limit the spread of pathogens [24,25], and significantly influence host phenotypes [26] and behavioral ecology [27]. In particular, gut microbes that exhibit high diversity and population diversity at the intra- and inter-specific levels [28] are crucial for intestinal homeostasis and host growth [29,30,31,32]. The diversity of the insect gut microbiome is regulated by numerous factors, such as host environment, diet, and age [33]. Notably, the diversity of symbionts in different insect tissues plays an important role in various physiological processes [34,35]. Generally, there are two modes of microbial transmission: horizontal and vertical [36]. Microorganisms in the reproductive organs of some insects are sex-specific; however, microbial exchange occurs through mating, increasing the similarity in the microbiota between female and male reproductive organs [37].

The host diet regulates the insect gut microbiota [38]. Imbalanced diets in hemipteran species influence the presence of different bacterial taxa, mainly, Actinobacteria, Bacteroidetes, Firmicutes, and Proteobacteria, within the host [39]. Heterotrophic Proteobacteria and/or Firmicutes dominate the gut microbiota of insects that feed on fruits or sap [40]. Gammaproteobacteria symbionts possess the biosynthetic capacity to synthesize and provide the host with essential nutrients, such as amino acids and vitamins [41]. Actinobacterial symbionts provide B vitamins to their insect host [42]. Shield bugs harbor a *Sodalis* strain as a core symbiont in anterior gut compartments that likely contributes to thiamine supplementation [43]. An obligate *Sodalis* symbiont of the slender pigeon louse was found to be maternally transmitted in bacteriocytes, supporting their host insect by supplementing nutrients and participating in digestion [44]. *Spiroplasma* species are abundant in the insect gut or hemolymph, where they have a large variety of commensal, pathogenic, or mutualist interactions with the host [45].

Phytophagous insect lineages have been used to test theories of evolutionary diversification [46]. Current research findings indicate that the intestinal symbiotic bacteria have remained stable at the species level in Pentatomidae [47]. However, a comparison of microbial lineages between evolutionarily related phytophagous and predatory insects has not been performed due to the complexity of symbiotic bacteria. Comparing the ecological niche of symbiotic bacteria in two insect species under laboratory conditions can help understand the influence of symbiotic bacteria on insect feeding and evolution and provide new insights to predict the performance of symbiotic bacteria involved in the life activities of insects.

The objective of the current study was to profile the symbiotic bacterial diversity in the tissues (salivary glands, gut, sperm, and ovaries) of two closely related bugs with different feeding habits, the phytophagous *H. halys* and the predatory *A. chinensis*, through 16S rRNA gene sequencing. We hope that the data presented herein will provide detailed insights into the potential relationships among symbiotic bacteria and insect feeding habits and reproduction, which may inform new biological control strategies.

## 2. Materials and Methods

### 2.1. Insect Cultivation

*A. chinensis* populations originated from adults collected in fields in Beijing, China. *A. chinensis* individuals were derived from a laboratory population maintained for over 70 generations. The insects were maintained in a rearing cage made from a plastic bottle (15.0 × 15.0 cm) and were fed *Antheraea pernyi* pupae.

*H. halys* populations originated from adults collected in fields in Sanming City in the Fujian Province of China. *H. halys* individuals were derived from a laboratory population maintained for over 35 generations and were fed corn and housed in boxes (34.5 × 23.3 × 16 cm).

The insects were reared under controlled conditions at 26 ± 1 °C with a 16 h light/8 h dark photoperiod and 65 ± 5% relative humidity. Adult specimens aged 5–7 days post-emergence were individually housed in 10 × 15 cm plastic bottles (a single insect/bottle) for subsequent experiments.

### 2.2. Sample Preparation

Prior to dissection, bugs of the two species were starved for 24 h to reduce the effects of the intestinal content. Virgin *A. chinensis* and *H. halys* were selected within 7 days of emergence; 70% ethanol was used for surface sterilization for 5 min, followed by rinsing thrice in sterile water. Next, they were immersed in pre-cooled phosphate-buffered saline (PBS) and dissected on ice with sterile forceps in a sterile environment. The dissected tissues and organs were rinsed in sterile PBS for 3 min and transferred into a 1.5 mL Eppendorf tube. All samples were immediately frozen in liquid nitrogen and stored at −80 °C until use. The gut (n = 92), salivary glands (n = 120), and reproductive organs (n = 92) were dissected carefully under sterile conditions to reduce contamination. The sample source, sample sex, sample name, and tissue source of each sample are shown in Table S1.

### 2.3. DNA Extraction and 16S rRNA Sequencing

Total genomic DNA was extracted from the samples using an OMEGA Soil DNA Kit (Omega Bio-Tek, Norcross, GA, USA), following the manufacturer’s instructions. The quality and integrity of the extracted DNA were determined using agarose gel electrophoresis, and DNA quantification was performed using a NanoDrop NC2000 spectrophotometer (Thermo Fisher Scientific, Waltham, MA, USA).

The forward primer 338F (5′-ACTCCTACGGGAGGCAGCA-3′) and reverse primer 806R (5′-GGACTACHVGGGTWTCTAAT-3′) were used for polymerase chain reaction (PCR) amplification of the V3–V4 region of the bacterial 16S rRNA gene. We incorporated 7 bp long sample-specific barcodes into the primers for multiplex sequencing. The PCR reaction (25 μL) included 5× buffer, 5 μL, 2.5 mM dNTPs, 2 μL, 5 U/μL Fast pfu DNA polymerase, 0.25 μL, 1 μL of the DNA template, 1 μL each of the forward and reverse primers (10 μM), and ddH_2_O to make up the final volume. The thermal cycling conditions were an initial 5 min of denaturation at 98 °C, followed by 25 cycles of rapid denaturation (30 s at 98 °C), annealing for 30 s at 53 °C, extension for 45 s at 72 °C, and a final extension for 5 min at 72 °C. The PCR amplicons were quantified using a Quant-iT PicoGreen dsDNA assay kit (Invitrogen, Carlsbad, CA, USA) and purified using Vazyme VAHTSTM DNA clean beads (Vazyme, Nanjing, China). After the individual quantification step, the purified amplicons were pooled, and sequencing was performed using the Illumina NovaSeq PE250 platform.

The original data were in FASTQ format and were analyzed using QIIME2 version (2024.10.1) and the recommended settings (https://docs.qiime2.org/2019.4/tutorials/, accessed on 25 July 2024). The resulting sequence was processed using DADA2 [48]. Amplicon sequence variants (ASVs) were obtained based on 100% sequence similarity. Using the SILVA database, characteristic ASV sequences were compared to reference sequences to acquire taxonomic information corresponding to each ASV [49]. ASVs with abundance values < 0.001% (1/100,000) of the total sample were removed for subsequent analyses.

### 2.4. Data Analysis

Alpha diversity was analyzed using the Shannon, Chao1, and Simpson indices, which are sensitive to high-abundance bacteria and can be computed using QIIME2 (2024.10.1). Beta diversity determined by principal coordinate analysis (PCoA) was calculated by computing the Bray–Curtis distance. Permutational multivariate analysis of variance (PERMANOVA) was performed using the R package ‘vegan’ (v2.6-4) [50]. Prediction of microbial function and pathways in the Kyoto Encyclopedia of Genes and Genomes (KEGG) database based on the 16S rRNA sequence data was performed by Phylogenetic Investigation of Communities by Reconstruction of Unobserved States (PICRUSt2). Diagrams were visualized using the R package (v2.6-6.1). Student’s *t*-test was used to assess statistical significance (*p* < 0.05 indicated statistical significance).

## 3. Results

**Figure 1:**
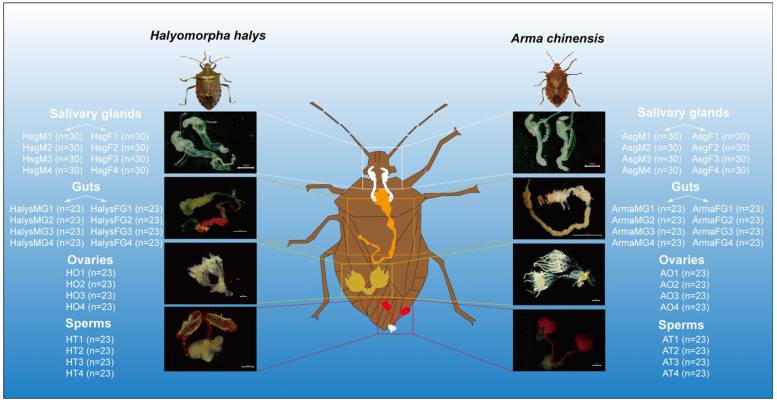
The datasets generated in these experiments were derived from 40 samples (four tissue types from two species). The collected tissues included the salivary glands, gut, sperm, and ovaries. Each experimental group included four biological replicates, and each replicate was formed by pooling tissue samples from several insects to minimize interindividual variation.

A description of the microbiota diversity in the salivary glands, reproductive organs, and gut of closely related bugs with different feeding habits was obtained through a 16S rRNA gene sequencing-based approach. An overview of the experimental design is shown in Figure 1.

### 3.1. Overall Distribution of Bacteria Within Different Organs

**Figure 2:**
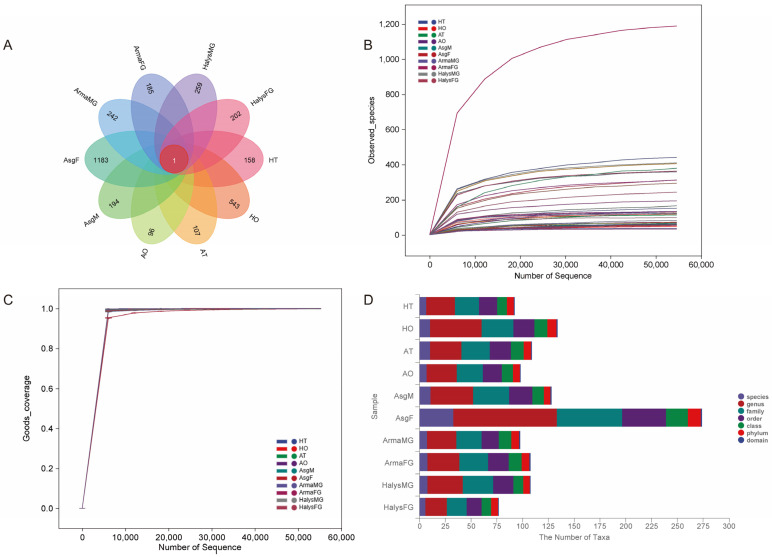
Sequencing information for the symbionts in *Arma chinensis* and *Halyomorpha halys*. (**A**) Venn diagram of the amplicon sequence variant (ASV) distribution. Rarefaction curve (**B**) and Goods’ coverage (**C**) of the observed species. (**D**) Taxon number statistics of the sequenced samples from the different organs of *A. chinensis* and *H. halys*. HT: testis of *H. halys*; HO: ovary of *H. halys*; AT: testis of *A. chinensis*; AO: ovary of *A. chinensis*; AsgM: salivary glands of male *A. chinensis*; AsgF: salivary glands of female *A. chinensis*; ArmaMG: gut of male *A. chinensis*; ArmaFG: gut of female *A. chinensis*; HalysMG: gut of male *H. halys*; HalysFG: gut of female *H. halys*.

A total of 4,574,879 raw tags and 3,845,705 valid tags were obtained from the tissue samples of 10 groups of *A. chinensis* and *H. halys* by analyzing the 16S rRNA amplicon sequences (Table S2). The length of the valid sequences was mostly distributed from 405 to 430 bp. A total of 7381 ASVs were clustered, comprising 14 phyla, 62 classes, 148 orders, 1983 families, 4266 genera, and 781 species (Table S3). Among the ASVs, only ASV-1305 was shared by the 10 tissue groups (Figure 2A). A high Good’s coverage index (>99%) indicated that the sequence depth adequately represented the majority of the ASVs present in all the tissue samples obtained (Figure 2B,C). A comparison of the statistical maps of the number of taxonomic units showed no significant differences in the microbial diversity of the tissues and organs of the two species at the taxonomic level (Figure 2D).

**Figure 3:**
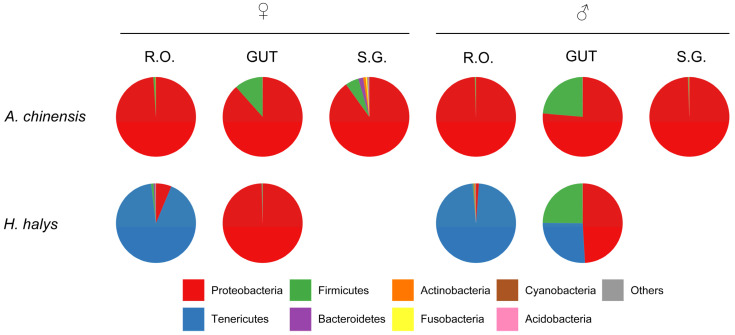
Percentage of amplicon sequence variants (ASVs) at the phylum level in the different organs of *Arma chinensis* and *Halyomorpha halys*. R.O.: reproductive organs; GUT: gut; S.G.: salivary glands; ♀: female; ♂: male.

Proteobacteria was the most extensively distributed phylum; Firmicutes was more commonly found in the gut of the two bugs, and Tenericutes primarily dominated the reproductive organs of *H. halys*. There were marked differences in the composition of the intestinal microbiota of *H. halys* between male and female adults (Figure 3). Previous proteomics research indicated that no bacterial proteins were present in the watery saliva or salivary sheaths of the salivary glands of *H. halys* [51]. In the present study, we also did not detect bacteria in the salivary glands of *H. halys*.

### 3.2. Microbiota Composition of the Reproductive Organs

**Figure 4:**
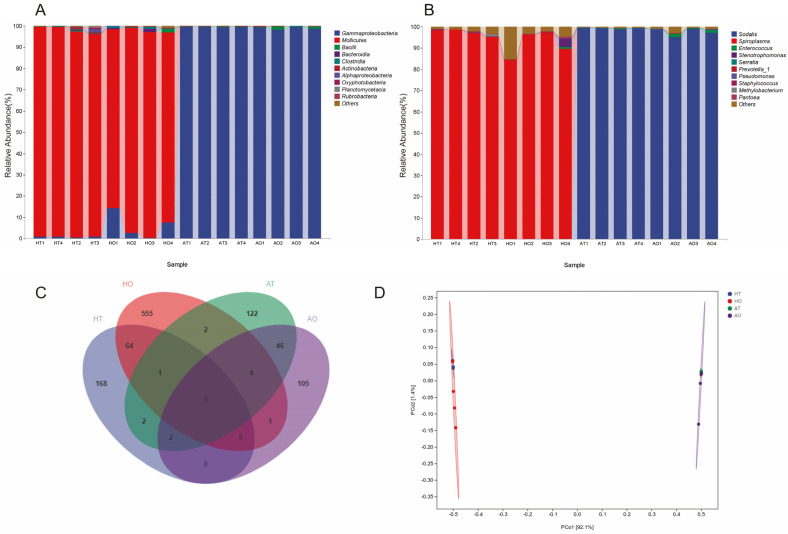
Top 10 dominant bacteria in the internal reproductive systems of *Arma chinensis* and *Halyomorpha halys* at the class (**A**) and genus (**B**) levels. (**C**) Venn diagram of the adult reproductive system samples. (**D**) PCoA analysis of the sequenced samples from the reproductive organs of the two bugs. HT: testis of *H. halys*; HO: ovary of *H. halys*; AT: testis of *A. chinensis*; AO: ovary of *A. chinensis*.

Upon categorization based on class, Mollicutes and Gammaproteobacteria were found in the reproductive organs of *H. halys*. The relative abundance of Gammaproteobacteria in *A. chinensis* was >98% (Figure 4A). At the genus level, *Spiroplasma* (>92%) was dominant in *H. halys*, while *Sodalis* (>97%) was dominant in *A. chinensis* (Figure 4B). To identify the shared or unique species in different samples, community analysis was performed using a Venn diagram (Figure 4C). *H. halys* testes contained 240 ASVs, while its ovaries contained 630 ASVs, of which 28 were shared. In contrast, *A. chinensis* testes contained 182 ASVs, the ovaries contained 161 ASVs, and 55 ASVs were shared. The reproductive organs of the two bugs shared three ASVs, namely, *Lactobacillus*, unclassified *Enterobacteriaceae*, and *Serratia*. PCoA showed marked differences between the microbial compositions of the reproductive organs of the two species (Figure 4D).

### 3.3. Microbiota Composition in the Gut

**Figure 5:**
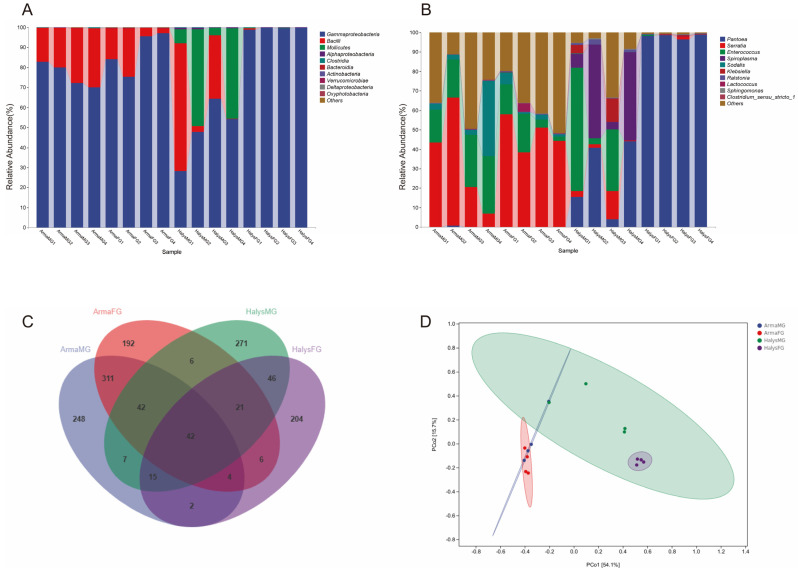
Top 10 dominant bacteria in the gut of *Arma chinensis* and *Halyomorpha halys* at the class (**A**) and genus (**B**) levels. (**C**) Venn diagram of the adult gut symbiotic bacteria. (**D**) Principal coordinate analysis of the sequenced samples from the gut of the investigated species. ArmaMG: gut of male *A. chinensis*; ArmaFG: gut of female *A. chinensis*; HalysMG: gut of male *H. halys*; HalysFG: gut of female *H. halys*.

The intestinal microbiota composition in *H. halys* showed sex specificity, while that in *A. chinensis* did not (Figure 5A). Regarding class diversity, Gammaproteobacteria were dominant in the female *H. halys* intestine (>99%), while there were three dominant bacteria in the male intestine: Gammaproteobacteria (M: 49%), Mollicutes (M: 26%), and Bacilli (M: 25%). Gammaproteobacteria (M: 76%, F: 88%) and Bacilli (M: 23%, F: 11%) were the dominant bacteria in the intestinal tract of *A. chinensis*. The dominant genus in the intestinal bacterial community of female *H. halys* was *Pantoea* (>98%), and those in the intestinal tissue of the males were *Pantoea*, *Spiroplasma*, *Enterococcus*, *Serratia*, *Klebsiella*, and *Ralstonia* (>1%). The most abundant intestinal bacteria in *A. chinensis* were *Serratia* (M: 34%, F: 48%), *Enterococcus* (M: 23%, F: 10%), *Sodalis* (M: 12%, F: 3%), and *Lactococcus* (M: 0.04%, F: 1.1%) (Figure 5B). Male *H. halys* intestine contained 450 ASVs, while the female intestine contained 340 ASVs; 124 ASVs were shared. Male *A. chinensis* intestine contained 671 ASVs, while the female intestine contained 624 ASVs; 399 ASVs were shared. Notably, 42 ASVs were shared between *H. halys* and *A. chinensis* (Figure 5C). PERMANOVA showed significant differences in the community structure of the intestinal microbiota between the two bugs with different feeding habits (PERMANOVA *R*^2^ = 0.66205, *p* = 0.001) (Figure 5D).

### 3.4. Microbiota Composition of the Salivary Glands

**Figure 6:**
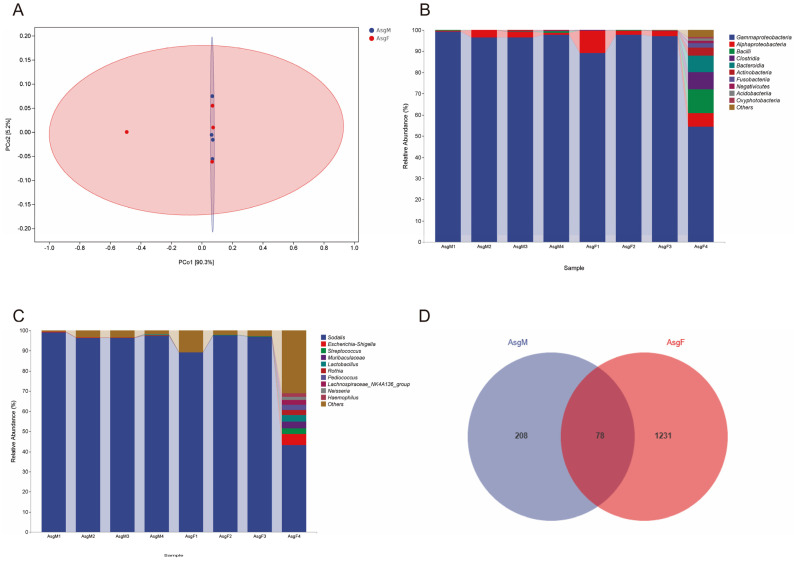
Principal coordinate analysis of the sequenced samples from the salivary glands of *Arma chinensis* (**A**). Top 10 dominant bacteria in the salivary glands at the class (**B**) and genus (**C**) levels. (**D**) Venn diagram of the salivary gland samples. AsgM: salivary glands of male *A. chinensis*; AsgF: salivary glands of female *A. chinensis*.

A similar microbiota was found in the salivary glands of *A. chinensis* individuals of different sex (PERMANOVA *R*^2^ = 0.13591, *p* = 0.801) (Figure 6A). Gammaproteobacteria were predominant (M: 98%, F: 85%), followed by Alphaproteobacteria (M: 2%, F: 5%) (Figure 6B). An analysis at the genus level indicated that the salivary glands of *A. chinensis* were primarily colonized by *Sodalis* (>82%) (Figure 6C). The salivary glands of female and male *A. chinensis* shared 78 ASVs (Figure 6D).

### 3.5. Prediction of the Function of the Variable Tissue Microflora in A. chinensis and H. halys

**Figure 7:**
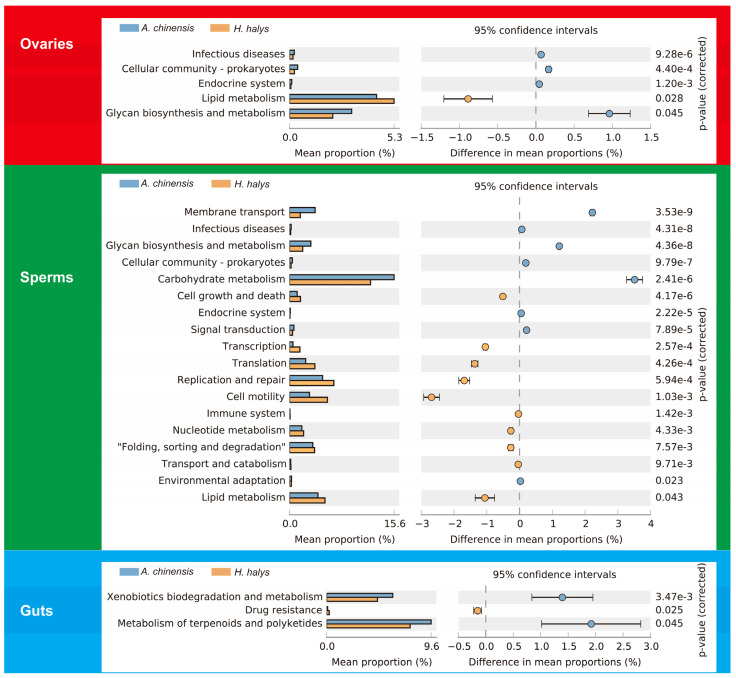
PICRUSt2 (v2.5.3) (https://github.com/picrust/picrust2, accessed on 20 August 2024) analysis was used to predict the KEGG pathways for *Arma chinensis* and *Halyomorpha halys* microbiota. The left-hand bar charts illustrate the average proportion of each KEGG pathway, while the right-side dot plots highlight the differences in average proportions between the two specified groups. *p*-values were calculated using the *t*-test.

PICRUSt2 software (v2.5.3) (https://github.com/picrust/picrust2, accessed on 20 August 2024) was used to predict bacterial function based on the 16S rRNA amplicon sequencing results to understand the importance of symbiotic bacteria in the two species of bugs with different feeding habits. The predicted results showed marked differences in bacterial function between *A. chinensis* and *H. halys* (Figure 7). KEGG analysis revealed that the “lipid metabolism” pathway was markedly more enriched (*p* < 0.05) in *H. halys* ovaries, while “glycan biosynthesis and metabolism” was significantly more enriched in *A. chinensis* ovaries. Notably distinct from that in the ovaries, the function of the sperm microflora exhibited marked variations between the two species. For example, “replication and repair”, “cell motility”, “lipid metabolism”, and “translation” were significantly more enriched in *H. halys* sperm, while “carbohydrate metabolism”, “membrane transport”, and “glycan biosynthesis and metabolism” were significantly more enriched in *A. chinensis* sperm. The intestinal microbial functions in *A. chinensis* were mainly “xenobiotic biodegradation and metabolism” and “metabolism of terpenoids and polyketides”, in contrast with those of *H. halys*.

## 4. Discussion

Microbial diversity in insects at different stages of development varies [52]. This study evaluated the diversity in microbiota composition in the organs of individuals (3–5 day-old virgin adults from a laboratory population maintained for more than 30 generations) of two species native to China. The Shannon indices of *A. chinensis* gut, salivary glands, sperm, and ovaries were significantly higher than those of the respective *H. halys* organs (Table S4), suggesting that the microbial communities in the digestive and reproductive organs of *A. chinensis* have a higher species diversity. These differences may reflect a more complex diet and reproduction for *A. chinensis*.

Some symbionts are designated as reproductive manipulators and alter the host reproduction in order to increase their likelihood of vertical transmission to the next host generation [36]. The rationale behind reproductive manipulation is the vertical transmission of endosymbionts. In tsetse flies, *Wolbachia* mainly resides in reproductive tissues and is maternally transmitted from generation to generation through trans-ovarian transmission [53]. *Sodalis*, in tsetse flies, is sexually transmitted from males to females and subsequently passed on vertically to the offspring [54]. The testes are the principal components of the reproductive system in male insects. Bacterial communities in the testes of some insects such as *Platypleura kaempferi* and *Bactrocera minax* have been identified [55,56]. In the testes of tsetse flies, the absence of symbionts depresses the activity of genes involved in the metabolic apparatus of male reproduction-related processes, such as sperm production, motility, and function. Conversely, the absence of symbionts reportedly activates gene expression in the testes [57]. In addition, variation in male testes microbial diversity correlates with male sperm viability, which contributes to male fitness in *Teleogryllus oceanicus* [58]. Our study revealed that *Sodalis* was the prevalent genus inhabiting the reproductive organs of *A. chinensis*. In contrast, *Spiroplasma* primarily colonized the sperm, ovaries, and intestinal tract of *H. halys*, indicating the possibility of vertical transmission of this bacterial species. In fact, all of the known defensive microbes, including strains of *Spiroplasma* [59], are vertically transmitted. Therefore, *Spiroplasma* in *H. halys* may manipulate its host to promote its reproduction, like in the parasitoid wasp *Lariophagus distinguendus* [60], or protect its host against infection by natural enemies [55].

Some studies have shown that PCR analysis of *H. halys* from Japan did not detect *Spiroplasma* [61], possibly due to differences in the diets of the sampled populations. According to the results of PICRUSt2 (v2.5.3) (https://github.com/picrust/picrust2, accessed on 20 August 2024) analyses, the functional differences between *Sodalis* and *Spiroplasma* are mainly reflected in the fact that *Sodalis* is more involved in carbohydrate and sugar metabolism, while *Spiroplasma* is more involved in lipid metabolism and biosynthesis. A pangenome analysis showed the core genes shared by all *Sodalis* strains, including genes for crucial cell functions, mainly, amino acid and carbohydrate metabolism and cell wall/membrane biosynthesis [62]. In *Drosophila*, females infected with *Spiroplasma* are less fecund and produce fewer eggs, which may be a consequence of nutritional competition between the fly and the bacterium for metabolically important free (hemolymph-borne) lipids [63].

This study demonstrated that, in addition to the feeding habits, sex is an important factor influencing the intestinal microbial composition. For instance, under the same feeding conditions, Proteobacteria accounted for 37.53% of ASVs in the gut of female *H. halys* and 42.49% in that of males. Consistent with previous research findings, microbial diversity was lower in the gut of female *H. halys* than in that of males [64]. The intestinal microbes of female *H. halys* primarily consist of *Pantoea*, which is vertically transferred to the offspring and provides essential nutrients that are unavailable in plant sap [43]. The intestinal microbiome diversity in male and female *A. chinensis* was comparable: Gammaproteobacteria and Bacilli accounted for the largest proportion of microbes in *A. chinensis* intestine. As predatory insects lack dominant bacteria and obligate symbionts [65], *Serratia*, *Enterococcus*, and *Sodalis* in *A. chinensis* intestines may significantly influence the prey. *Serratia* serves as an invaluable model for evaluating the evolutionary trajectories of bacterial acquisition in insects because it has diverse strains [66]. *Serratia* includes a great diversity of strains associated with very distinct biological features and reflecting various functions that bacteria can share with their aphid hosts [67,68,69]. The strains studied in the subfamily Aphidinae have been first described as intracellular facultative partners because they can invade the host cells and can be associated with protective phenotypes (protection from environmental heat stress and parasitoids) [70,71,72]. The *Serratia symbiotica* strains associated with the subfamilies Lachninae and Chaitophorinae are intracellular symbionts involved in co-obligate associations, compensating for some metabolic capacities lost by their ancient obligate symbiont [73]. In this study, *Serratia*, the most abundant intestinal bacterium in *A. chinensis*, accounted for 34% of the intestinal bacteria in males and 48% in females, while in *H. halys*, it accounted only for 4.94% of the intestinal bacteria in males and 0.49% in females. A recent study showed that the interaction of pathogenic *Serratia* bacteria with harmonine in harlequin ladybird conferred an interspecies competitive edge [74]. The relatively high abundance of *Serratia* in *A. chinensis* indicates that this symbiotic bacterium may play some special roles in this predatory bug and is worthy of further study.

In this study, despite multiple attempts, PCR amplification of 16S rRNA sequences from the salivary glands of *H. halys* was unsuccessful. Consequently, the bacterial communities of the salivary glands could not be compared between the two insect species. A previous proteomics study also indicated the absence of bacterial proteins in the watery saliva or salivary sheaths produced by *H. halys* salivary glands [51]. Further investigations are needed to confirm the presence or absence of symbionts in the salivary glands of this species. This study found that the dominant genus in *A. chinensis* salivary glands was *Sodalis*, which differs from observations in triatomines [75], possibly due to differences in the diets of these two types of insects.

Several studies on the differences between phytophagous and predatory bugs focusing on morphology [76,77,78], mitochondrial phylogenomics, and chromosome-level genome [79,80,81,82] have been reported. A difference in insecticide susceptibility between phytophagous and predatory bugs has been demonstrated [83]. Investigation on the gut microbiota of true bugs with different living habitats and feeding habits showed the impact of environmental habitats and diets on the diversity of the gut bacterial community [84]. Given the megadiversity of true bugs, investigations into their association with symbiotic bacteria have been mainly restricted to phytophagous insects, and there is a lack of a comprehensive characterization of the bacterial communities associated with their feeding, digestive, and reproductive organs. As this study is one of few focusing on comparing symbiotic bacteria between two closely related species with different feeding habits, our results provide additional insights into the relationship between microbiome composition and insects’ feeding habits and reproduction.

Augmentative biological control is dependent upon the production of large numbers of natural enemies of high quality. The search for suitable artificial diets for predatory pentatomids is ongoing. Symbionts in the salivary glands and gut may affect the performance of predators on artificial diets. Probiotics and prebiotics play import roles in the maintenance of health in mass-reared insects [85], including synthesizing some nutrients that are lacking in natural foods and secreting digestive enzymes for food digestion [86,87]. Our current data provide a theoretical basis for the screening of synergistic probiotics in artificial diets and the upgrading of artificial diets. Bacterial species present within an insect organ can exhibit mutualism or commensalism or could even be pathogenic. Insect pathogenic bacteria and their derived products represent the active substances of various biopesticides. Significant cases include the entomopathogenic nematode symbionts *Photorhabdus* spp. and *Xenorhabdus* spp., *Serratia* species, *Yersinia entomophaga*, *Pseudomonas entomophila*, and the recently discovered Betaproteobacteria species *Burkholderia* spp. and *Chromobacterium* spp. [88]. In addition, the obligate reliance of many insects on their microbial partners provides a potential target for the biological control of devastating agricultural pests. As such, numerous studies have examined the importance of the associated microorganisms to host fitness and feeding ecology in an effort to manipulate these partnerships and render insect pests more vulnerable to broad-scale measures of population control by targeting their bacterial symbionts. Prevention of symbiont acquisition through surface sterilization of *H. halys* eggs results in nymph developmental delays in the first generation [89]. The understanding of symbiont transmission and its effect on host fitness will allow for the development of a new pest control strategy based on symbiont disruption.

## 5. Conclusions

This study confirmed that the microbiomes of two insect species with different feeding habits differed significantly in feeding, digestion, and reproduction organs. The results showed that the predatory *A. chinensis* has a more complex bacterial diversity than the phytophagous *H. halys*. Our current data provide new insights for developing synergistic probiotic-based artificial diets for predators and symbiont disruption-based pest control strategies. Moreover, the bacteria of the testes may serve as a key factor in the genetic evolution of both species.

## Data Availability

The raw data can be found in the NCBI SRA database (accession numbers: PRJNA1163309) on the website: (http://www.ncbi.nlm.nih.gov/bioproject/1163309, accessed on 27 October 2024).

## Supplementary Materials

The following supporting information can be downloaded at: https://www.mdpi.com/article/10.3390/insects16020146/s1, Table S1: Meta-table of the samples in the dataset; Table S2: Statistical table of sequencing volume per sample; Table S3: The ASV counts and taxonomy. Table S4: Bacterial diversity and richness in salivary glands, gut, and reproductive organs of *Arma chinensis* and *Halyomorpha halys*.
